# A straight pin foreign body in a child: ingested or aspirated?

**DOI:** 10.1186/s40064-016-3335-6

**Published:** 2016-10-01

**Authors:** Xicheng Deng, Jinghua Wang, Renwei Chen, Peng Huang, Pingbo Liu, Xinyou Luo

**Affiliations:** 1 Department of Cardiothoracic Surgery, Hunan Children’s Hospital, No. 86 Ziyuan Road, Changsha, 410007 Hunan China; 2 Department of Otorhinolaryngology, Hunan Children’s Hospital, Changsha, Hunan 410007 China

**Keywords:** Foreign body, Airway, Surgery, Child

## Abstract

**Background:**

Though foreign body (FB) aspiration or ingestion is not uncommon in children, a straight pin as the culprit FB is rarely seen. The nature of such a FB makes it sometimes difficult to diagnose and deal with, especially in children.

**Case report:**

Here we present such a case who was initially misdiagnosed with FB ingestion but turned out to be an aspiration case. Moreover, its remote location from the hilum made a more invasive surgical retrieval inevitable. A thoracotomy was finally performed to retrieve the pin. And the postoperative course was uneventful.

**Conclusion:**

For pediatric FB cases, especially in such a case, it is very important to diagnose timely and accurately. A multidisciplinary team approach would facilitate prompt and accurate diagnosis and potentially simplify treatment.

## Background

Foreign bodies, either aspirated or ingested, are very common in children (Cheng and Tam 1999; Zitzmann et al. 1999; Louie and Bradin 2009; Lorenz 1983; Eren et al. 2003). The most usual foreign bodies include nuts, buttons and toy parts, most of which can be dealt with non-surgical measures (Louie and Bradin 2009; Cheng and Tam 1999). A straight pin, however, is rarely seen as the culprit. The nature of this kind of foreign body (FB) and its very low incidence make it difficult to diagnose and treat.

We present here a rare pediatric inhaled straight pin case mimicking a swallowed one and discuss its diagnosis and treatment.

## Case

**Fig. 1:**
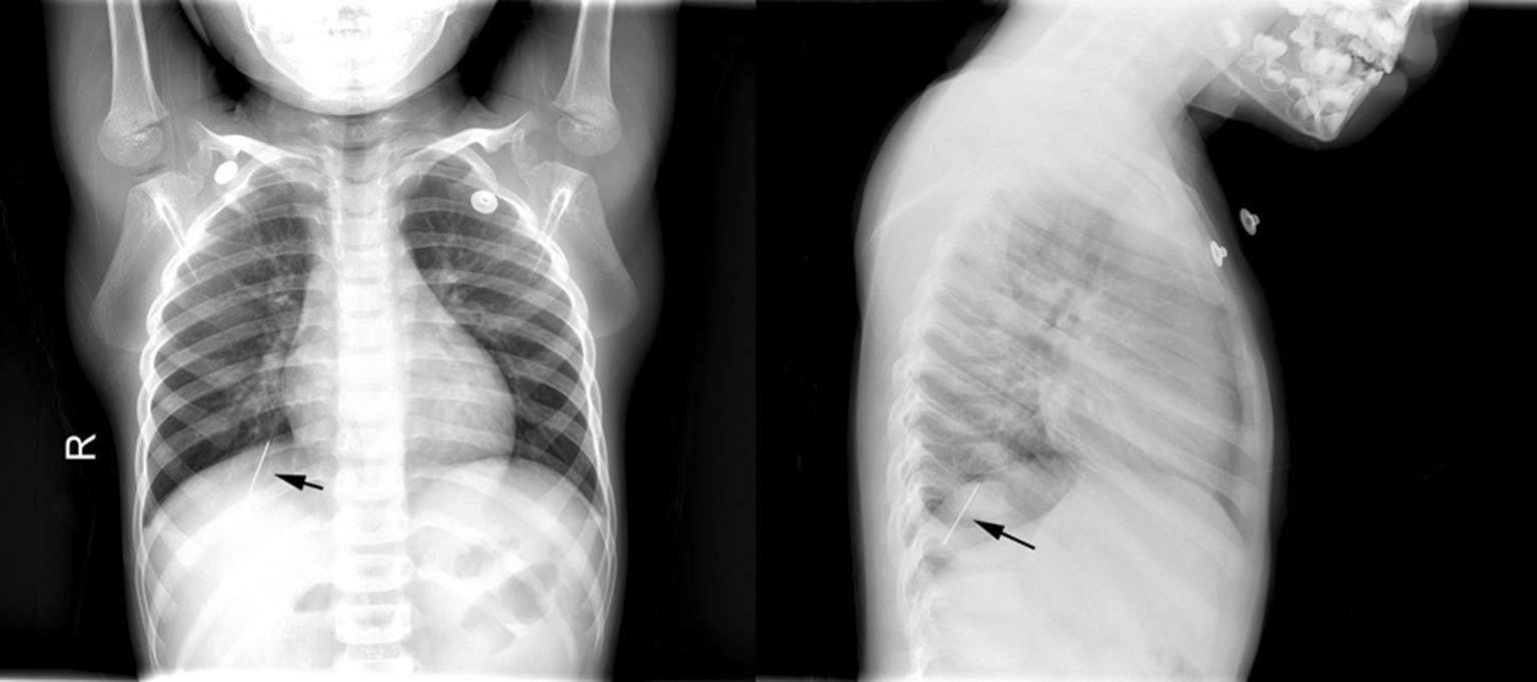
*Black arrows* indicate the pin

**Fig. 2:**
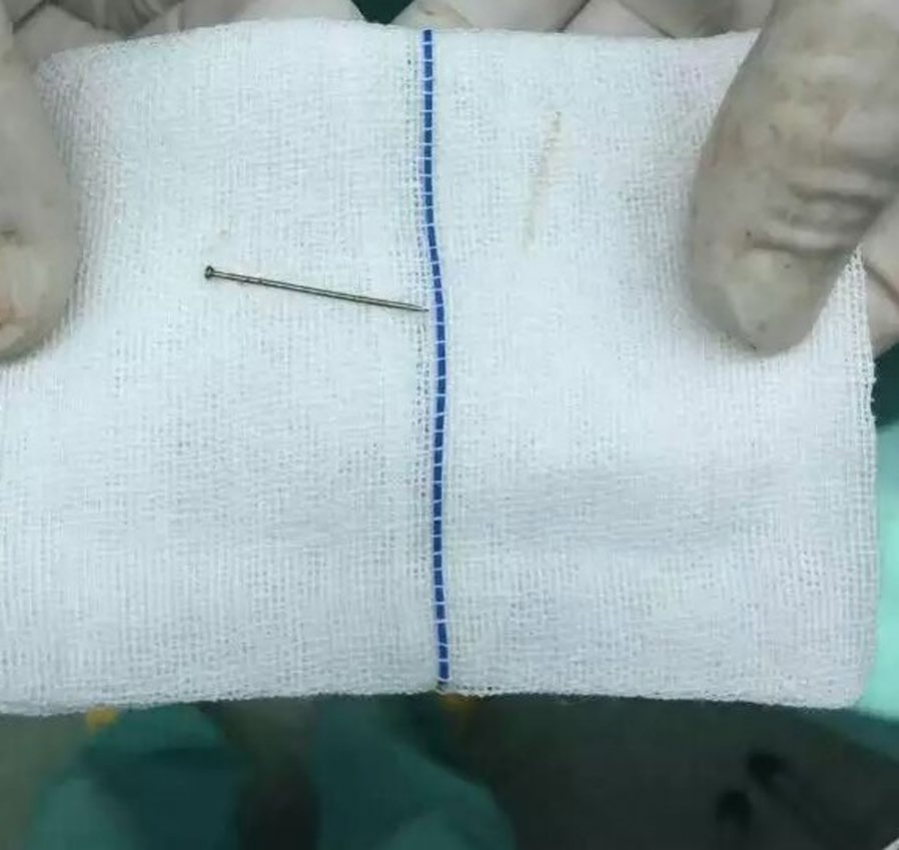
Retrieved pin

A formal written consent has been obtained from the parents regarding publication of the following child’s personal and medical information. A 6 years old boy was sent by his parents to the emergency department of our hospital for “having swallowed an iron nail” 15 h ago though his parent did not witness the accident on site. He felt no pain or discomfort, nor had he had vomit or fever. There was no evident positive finding on physical examination. An upright abdomen and chest X-ray revealed “a nail FB” in gastrointestinal (GI) tract. The patient was admitted to general surgery department for further treatment. The following day after admission, a second chest X-ray found it in its original place. However, with a high index of suspicion, the radiologist performed a fluoroscopy and found the FB, likely a straight pin, was actually in the posterobasal segment of the right lung (Fig. 1). The boy then underwent a computed tomography (CT) scan to further confirm a diagnosis of right lung FB, after which the child was transferred to our department. An immediate rigid bronchoscopy failed to approach the pin since it was too far away from the hilum. As such, we then performed an emergency thoracotomy. The right chest was opened through a posterolateral incision in the eighth intercostal space. The pin was sensed by hand-touching and pinched and held with forceps. A hole in the lung was made against the pin using cautery before the pin was completely and smoothly taken out (Fig. 2). The postoperative course was uneventful and the boy was discharged on day 7.

## Discussion

A literature search in PubMed was conducted using a word combination of “pin”, “FB” and “child”. We found no cases in the English-language literature of an airway straight pin in the child as the present one we report here. We discuss its diagnosis and management as follows.

Foreign bodies, either ingested or aspirated, are very common in children (Louie and Bradin 2009; Zerella et al. 1998; Cheng and Tam 1999). A straight pin, however, is rarely seen as the culprit. Though, based on history, symptoms and imaging workup findings, most cases can be diagnosed in a timely fashion without much difficult, it remains important for emergency doctors to differentiate an airway FB from a GI FB in some cases.

With regards to history, FB patients are frequently brought to see a doctor after being witnessed or the children report by themselves. Yet in patients who present due to complications or whose diagnosis is made incidentally, the history may be negative. Furthermore, it not uncommon that as babies or young kids can not present themselves at all or very well, it’s hard to judge from present history, which may occasionally be complicated by absence of their guardians when such an event occurs. Sometimes, parents’ preemptive present history narrative may mislead diagnosis as shown in present case.

The spectrum of symptoms for acute FB cases range from asymptomatic state to choking, neck/throat/abdominal pain, cough, etc. to dyspnea. Some of them (pain, choking) are shared by aspirated and ingested FB, yet some are unique (FB sensation, dysphagia for ingestion and dyspnea for aspiration) (Louie and Bradin 2009). Symptoms can give clue to the diagnosis, but in asymptomatic patients, history and imaging examination may provide enough information.

Evaluation of a patient suspected of having a FB should generally include plain films covering the whole airway or the GI system. For radiolucent objects, contrasts are to be used for identification. Though, in most cases, a radiographic film can clearly show the location of the FB, it should be carefully interpreted to minimize misdiagnosis. In our case, the pin was lodged deeply in peripheral lung and it looked even below the right diaphragm on the initial posteroanterior abdomen and chest X-ray, as opposed to literature (Ilan et al. 2012), which present cases with a pin high above the diaphragm. Combined with a history of “having swallowed a nail” and negative symptoms, that was why he was initially misdiagnosed with a GI FB. In suspicion of the diagnosis during the second chest X-ray workup, the radiologist performed fluoroscopy at once. Bronchoscopy may be considered as the next diagnostic and therapeutic method. However, it is generally regarded as more invasive than a CT scan, though with ionized radiation for the latter one. Requiring anesthesia for bronchoscopy is another factor that has impact on decision making, especially in the setting of a potential misdiagnosis. What’s unique for this case is there was no witness of history or positive symptoms, and it’s not clear if it was an airway or GI tract FB. As such, CT scan, which is generally not recommended in FB diagnosis, was performed. For an airway FB, besides plain X-ray film, bronchoscopy is the most commonly used modality for diagnostic and therapeutic purposes. Rigid bronchoscopy has been considered method of choice, especially in symptomatic cases (Martinot et al. 1997), yet some recent studies have also shown flexible bronchoscopy can be performed safely with minimal risks and complications for diagnosis and treatment (Swanson et al. 2002; Dikensoy et al. 2002; Ramirez-Figueroa et al. 2005; Berraies et al. 2014). In some specific cases, it is even superior to rigid bronchoscopy (Aslan et al. 2013). The failed recovery of the pin by rigid bronchoscopy in our case is because the FB was too peripherally lodged and a rigid bronchoscope was too large to reach. After consultation with otorhinolaryngologists and pulmonologists, flexible bronchoscopy was deemed inappropriate with regard to its location. Decision was then made to go forward with a thoracotomy.

As far as the nature of an FB is concerned, the majority of foreign bodies in pediatric patients are toy parts, small pieces of food or others, like nuts, seeds and buttons. (Schneider 1982; Eren et al. 2003) For an airway FB, it is not common to find a pin in a young child, (Eren et al. 2003) though it is reported frequently that in Muslim women, turban pin aspiration is quite often seen (Ilan et al. 2012; Ragab et al. 2007). According to literature, most of these turban pin can be retrieved by bronchoscopy since they are usually not very distant from the hilum (Ilan et al. 2012). We consider it may be because all these turban pins, different from the pin in our case, have a larger plastic pin head, which presumably stop them from moving further. By contrast, in our case, the pin head was metal, smooth and only slightly larger than the pin body. Also, there is a study (Ludemann and Riding 2007) from a western country reporting sharp FBs including pins, needles and a blowdart in adolescents with typical symptoms versus a preschooler with no positive presentation in our case.

The literature has reported involvement of interventional cardiologist during recovery for a peripherally lodged airway FB (Thatte et al. 2014). An inaccessible airway FB can ultimately results in chest open surgery (Harischandra et al. 2009). FB cases in the child should be managed with a multidisciplinary approach. It may, from diagnosis to treatment, involve emergency doctors, radiologists, otorhinolaryngologists, pulmonologists, gastroenterologist, and general and cardiothoracic surgeons. Timely diagnosis and treatment may be a key factor for the management of FB patients especially cases like the present one. Theoretically, once entering trachea, an airway FB can move further and deeper until it is stuck somewhere. In view of this, it is better to have the case managed by a capable hospital and team of physicians as soon as possible. Delayed presence or unnecessary transfer may provide time for an FB to drop further which makes an endoscopic retrieval impossible or even penetration of the airway. As emergency as such a case is, a team approach can make a faster and more accurate diagnosis. The time saved before intervention may potentially simplify treatment. Through working closely, it also provides best of care for these patients.

## Conclusions

Though foreign bodies, either ingested or aspirated, are very common in children, a straight pin in a child is not usually seen in clinical settings. Sometimes a FB could not be easily identified if it is ingested or aspirated. As emergency as such a case is, a team approach can make a faster and more accurate diagnosis and provides best of care for these patients.

## Abbreviations

FB: foreign body
CT: computed tomography
GI: gastrointestinal
